# Evaluation of Psychophysical Factors in Individuals with Frailty Syndrome Following a 3-Month Controlled Physical Activity Program

**DOI:** 10.3390/ijerph17217804

**Published:** 2020-10-25

**Authors:** Wioletta Dziubek, Weronika Pawlaczyk, Małgorzata Stefańska, Joanna Waligóra, Maria Bujnowska-Fedak, Joanna Kowalska

**Affiliations:** 1 Department of Physiotherapy, University School of Physical Education, 51-612 Wroclaw, Poland; wioletta.dziubek@awf.wroc.pl (W.D.); malgorzata.stefanska@awf.wroc.pl (M.S.); joanna.kowalska@awf.wroc.pl (J.K.); 2 Lower Silesia Oncology Center, 53-413 Wroclaw, Poland; 3 Department of Family Medicine, Wroclaw Medical University, 51-141 Wroclaw, Poland; joanna.waligora@student.umed.wroc.pl (J.W.); maria.bujnowska-fedak@umed.wroc.pl (M.B.-F.)

**Keywords:** frailty syndrome, physiotherapy, exercise, mood, BDI, STAI, SWLS, muscle strength

## Abstract

Background: The aim of the study was to compare the emotional state and strength-velocity parameters of patients with frailty and pre-frailty syndrome undertaking a 12-week training programme. Methods: The study was completed by 36 individuals, including 17 with frailty syndrome (FS) and 19 with pre-frailty syndrome (PFS). The age of the subjects ranged from 63 to 89 years, with a mean 69.2 years (±5.0). The Beck Depression Inventory (BDI), Spielberg’s State-Trait Anxiety Inventory (STAI), and Satisfaction with Life Scale (SWLS) were used. The strength of knee muscles was evaluated. The above tests were conducted at two time points: before the training sessions (T1); and after 12 weeks of regular training sessions (T2). Results: After completion of the training programme, statistically significant differences in BDI were observed between the PFS and FS groups (especially in somatic symptoms). Following the training, BDI values in the PFS group were significantly lower (fewer depressive symptoms) than in the FS group. The parameter values describing strength capacities of the lower limbs, both at T1 and T2, proved to be higher in the PFS group. Conclusions: In individuals with pre-frailty and frailty syndrome, the 3-month physical training programme improved the strength parameters of lower limb muscles. An improvement in mood and reduction in depressive symptoms were only observed in the group of subjects with pre-frailty syndrome. Rehabilitation programmes for people with frailty syndrome should include psychotherapeutic activities in addition to physical training in order to improve the psychophysical condition of patients.

## 1. Introduction

Advancing age is associated with decreased fitness and physical performance, a deterioration in psychophysical condition, decreased strength and muscle mass (sarcopenia) and impaired balance and neuromuscular coordination. Additionally, elderly people with a sedentary lifestyle suffer from bone weakness and increased susceptibility to injuries and fractures.

Frailty syndrome, otherwise known as weakness or fragility syndrome, is defined as a clinically recognised state of increased sensitivity of the body to endo- and exogenous stress factors due to reduced physiological reserves, resulting from a reduced capacity of various physiological systems [1].

The aetiology of frailty syndrome is not entirely understood. An international group of experts has defined frailty as a clinical state in which there is an increase in an individual’s vulnerability for developing increased dependency and/or mortality when exposed to a stressor [2]. Many interconnected negative stress factors are known to contribute to its development, causing a decrease in systemic reserves at the molecular, cellular and physiological levels, and exacerbating pre-existing pathologies [3].

Elderly people who have been diagnosed with frailty syndrome due to depleting functional organ reserves experience accelerated aging processes [3]. Deterioration of the body’s ability to react to stress, disruptions to homeostasis and impairment in regeneration function lead to a more severe disease course, which increases their susceptibility to adverse events such as falls, disability, hospitalisation and death [4]. Additionally, in people over 60 years of age with frailty syndrome, changes to the skeletal muscles such as a reductions in muscle mass and muscle contraction strength, nerve atrophy and slowing of the contractility of muscle fibres are very common. These changes are associated with sarcopenia, a reduction in muscle mass, and, consequently, a reduction in muscle strength. This is associated with a loss of motor units present in muscle fibres. This phenomenon occurs to a similar extent in both genders, with the most significant changes occurring in the lower limbs. Hormonal changes are also thought to have a destructive influence, with decreased hormone concentrations leading to reductions in muscle mass, strength and muscle function. In addition, a lack of physical activity is known to increase the risk of sarcopenia [5].

Changes in the musculoskeletal system may contribute to a loss of physical fitness, impaired balance and falls, leading to a loss of independence and difficulty performing simple and complex activities.

The most frequently used criteria to identify frailty syndrome were proposed by Fried et al. (2001) [6]. These criteria include five indicators: weight loss of more than 5 kg per year, reduced hand grip strength measured on a dynamometer, exhaustion assessed by the CES-D depression scale, reduced gait speed and reduced physical activity measured by the modified Minnesota Leisure Time Activity Questionnaire. The patient must meet three or more criteria for frailty syndrome (FS) to be recognised. If one to two criteria are met, a condition referred to as pre-frailty syndrome (PFS) is declared, which precedes the diagnosis of frailty syndrome [6].

A lack of physical activity is known to accelerate the aging process of the body and may lead to physical disability. Undertaking physical activity has a positive impact not only on physical fitness, but also on mental health, contributing to an improvement in mood and reduced levels of depression and anxiety [7].

The latest scientific reports on frailty syndrome suggest that it can be prevented and treated with regular physical activity [8,9]. According to Mazurek et al. (2018), health training activities positively influence each of the diagnostic criteria that are typical of frailty syndrome, such as weight loss, exercise intolerance, slowing down of gait, weakening of muscle strength and a subjective feeling of weakness and fatigue [10].

Very few scientific reports on this topic have focused on exploring such forms of training that would benefit patients with frailty syndrome at various stages of the disease, as well as to promote effective and scientifically proven physical activity among the elderly as a generally accepted intervention to prevent frailty syndrome [2,11].

### Study Aim

The aim of the study was to compare the emotional state and strength-velocity parameters of patients with frailty and pre-frailty syndrome undertaking a 12-week training programme. Specifically, we wanted to assess the emotional state of subjects with frailty and pre-frailty syndrome and determine whether a 12-week training programme improves their mood, and to investigate whether there is a relationship between the emotional state and strength-velocity parameters in the two groups of patients.

## 2. Material and Methods

### 2.1. Study Group

Studies were conducted at the Scientific Research Laboratory of the Department of Physiotherapy at the University of Physical Education in Wroclaw. Each participant was informed about the purpose and method of the study and about the possibility of withdrawing from the study at any stage. Participants provided informed consent to take part in the study. The study was approved by the Bioethics Committee of the University School of Physical Education in Wroclaw, Poland (reference no. 15/2020) and conducted in accordance with the Declaration of Helsinki.

Individuals with frailty and pre-frailty syndrome who met the following inclusion criteria qualified for the study: at least three out of five symptoms of frailty syndrome or one to two symptoms in the case of pre-frailty (according to Fried frailty index) confirmed by a doctor, no contraindications to the tests and trials, no participation in another rehabilitation programme, absence of dementia (MMSE > 24), and consent to participate in tests and trainings. Exclusion criteria were also adopted: contraindications to exercise tests and physical training, dysfunctions that make it impossible to perform tests and participate in trainings, less than 70% of training attendance.

The study was completed by 36 individuals, including 17 with frailty syndrome and 19 with pre-frailty syndrome. The age of the subjects ranged from 63 to 89 years, mean 72.1 years (±6.4).

**Figure 1:**
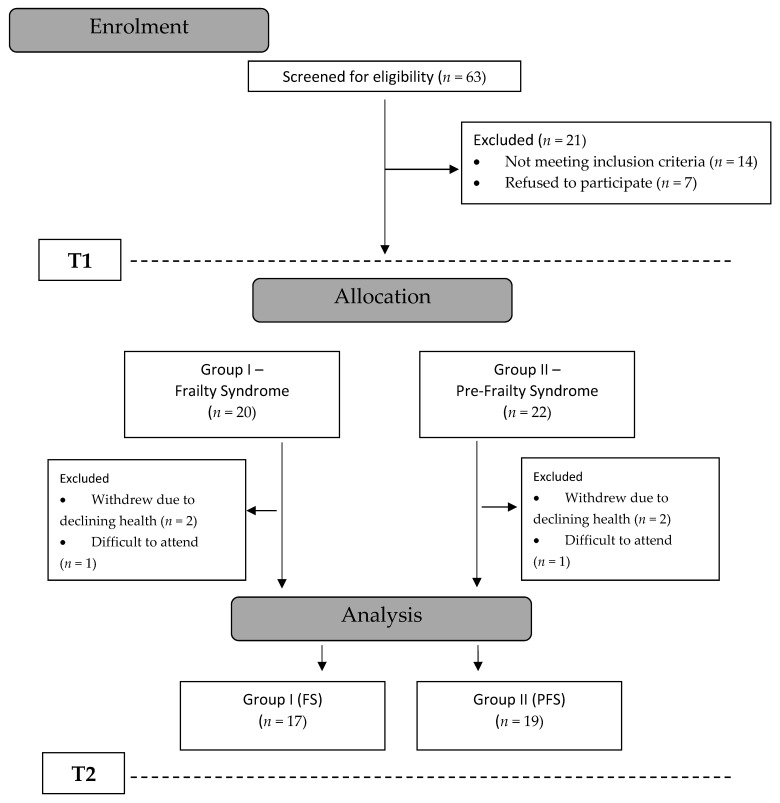
Design and flow of participants throughout the study.

Patients from both groups took part in regular training sessions (Figure 1).

### 2.2. Measurement Tools

The Beck Depression Inventory (BDI), Spielberg’s State-Trait Anxiety Inventory (STAI), and Satisfaction with Life Scale (SWLS) were used.

The BDI contains 21 items that relate to most significant symptoms of depression. The Polish version of the BDI is a translation of the original tool with very good psychometric properties similar to the original (Cronbach’s α was 0.95 for clinical trial and 0.93 for control group). The first 13 questions focus on cognitive-affective aspects and the remaining questions relate to somatic symptoms that accompany mood disorders. Scores of 0–11 points indicate no depressive disorders, while higher total scores indicate more severe depressive symptoms according to the applicable standards [12,13].

The SWLS measures an individual’s subjective sense of satisfaction with life. The higher the score, the more satisfied with life the respondent is. This study used raw results of the Polish standards, where scores of 5–17 indicate low satisfaction, 18–23 indicate average satisfaction and 24–35 represent high satisfaction with life. The psychometric properties of the Polish version are satisfactory and similar to the original [14,15].

The STAI was used to assess the level of anxiety as a state and as a trait. The subscale of anxiety as a state (X-1) is used to study the current mood of the respondent, while the trait anxiety subscale (X-2) illustrates how the assessed person usually feels. Measurement does not include somatic manifestations of anxiety. The criterion for dividing patients into subgroups of low and high levels of anxiety for the STAI (X-1) is a score of 44, and for STAI (X-2) it is a score of 46. The overall result for each of the two parts of the questionnaire ranges from 20 points, indicating mild anxiety, to 80 points, indicating very severe anxiety. The psychometric properties of the Polish version are similar to the original [16,17].

### 2.3. Examination of Force–Velocity Parameters

The right and left flexor and extensor muscles of the knee joint were evaluated. The following parameters were analysed: peak torque (Nm), total work (J), and average power (W). The measurements were made using a Multi Joint 4 dynamometer (Biodex, Shirley, NY, USA).

The subject performed flexion and extension tests in isokinetic conditions. Before the measurement, the attachment, seat and dynamometer were adjusted so that extension of the axis of rotation of particular joint was achieved. With appropriate stabilisation, the measurement began with the maximum flexion of the knee, performed fast and with the greatest possible strength. For angular velocity ω = 60°/s, five repetitions were performed [18].

The above tests were conducted at two time points: before the training sessions (T1); and after 12 weeks of regular training sessions (T2).

### 2.4. Training Sessions

The respondents exercised regularly twice a week for 60 min for 12 weeks. The exercises were carried out in the gymnasium of the Faculty of Physiotherapy of the University of Physical Education in Wroclaw. A single session consisted of a warm-up (10 min) and the main physical workout (about 40 min), followed by stretching, breathing and relaxation exercises (10 min). During the session, the subjects performed general fitness exercises, improving coordination and balance, as well as resistance exercises using Thera-Band with an individually adjusted load. Other sporting equipment was also used, including mats, gym rods and balls.

### 2.5. Statistical Analysis

The study group was characterised using descriptive statistics, including mean, standard deviation, median, minimum and maximum values, and numbers, confidence interval and percentages were used in the case of qualitative variables. The Shapiro–Wilk test was used to check for normal distribution of the data. Non-parametric tests were used for BDI, SWLS and STAI data (Wilcoxon test to compare two dependent groups and Mann–Whitney U test to compare two independent groups) and parametric tests for force–velocity parameters (Student’s *t*-test to compare two dependent groups and to compare two independent groups). Cohen’s d coefficient, pairs rank biserial correlation coefficient as well as Glass rank-biserial correlation coefficient were calculated to assess the magnitude of the effect of the observed relationships [19,20,21,22]. The calculations were carried out using STATISTICA 13.1. (StatSoft Polska, Kraków, Poland).

### 2.6. Results

The comparative analysis of both groups showed that the group of patients with PFS was significantly younger than those with FS (Table 1).

Before the commencement of training sessions (T1), depressive symptoms (BDI ≥ 12) were recorded in 16 respondents (44%), most of whom were in the PFS group (10 subjects, 53%). After 12 weeks of training (T2), 14 people (39%) still had depressive symptoms. However, at this time point, the number of people with depressive symptoms increased to 10 in the FS group (59%) (Table 2).

In the PFS group, a statistically significant decrease in the values describing depressive symptoms (BDI) was observed. The levels of both state anxiety (STAI X-1) and trait anxiety (STAI X-2) were also significantly decreased in this group. Similar trends were not observed in the group with frailty syndrome. Moreover, the level of life satisfaction (SWLS) did not change in either group. The observed relationships were confirmed by a low effect size (r_c_ ≤ 0.4) with no statistical significance and a medium to high effect in the case of statistically significant changes (r_c_ ≥ 0.55) (Table 3).

The peak torque (PT [Nm]), total work (TW [J]), and average power (aP [W]) were evaluated twice (T1 and T2) during flexion and extension of the knee joint under isokinetic conditions with 60°/s load. The results for these parameters observed after the completion of the training programme in both study groups were higher than the baseline values (Table 4).

Prior to the training programme, no significant differences in the level of depression and anxiety symptoms were observed between the groups. After completion of the training programme, statistically significant differences in BDI were observed between the PFS and FS groups (especially in somatic symptoms). Following the training, BDI values in the PFS group were significantly lower (fewer depressive symptoms) than in the FS group (Table 3 and Table 5).

The parameter values describing strength capacities of the lower limbs, both at T1 and T2, proved to be higher in the PFS group (Table 4 and Table 5). In the case of statistically significant differences between values obtained in the first and second study, the effect size was greater than 0.2 in both studied groups in most cases (Table 5). For statistically significant differences observed between the groups in both study 1 and 2, the effect size was greater than 0.72 (Table 5).

## 3. Discussion

Individuals with frailty syndrome are more susceptible to stress and exhibit a poorer psychophysical condition compared to their peers. It has been found that too little physical activity activates the so-called “cycle of weakness”, which further deteriorates fitness that is already hindered by the ageing process [10].

For the elderly, maintaining an active lifestyle and continuing daily activities are particularly important. A planned and systematic physical activity routine positively influences not only the individual’s physical health, but also their cognitive-emotional state. Many published studies have confirmed these findings [10,23,24,25,26]. According to Chris et al. (2017), physical activity shapes a personality, which can also indirectly influence the individual’s emotional state [27].

The training performed in this study had a clear impact on the emotional state of patients. In the group of patients with a pre-frailty syndrome, a significant improvement in mood and reduced anxiety were observed. This was in contrast to the group of patients with frailty syndrome, in whom a deterioration in mood and increase in anxiety symptoms were observed following the training, but this difference did not reach statistical significance. It is worth mentioning that, in the pre-frailty syndrome group, the number of depressive disorders was significantly decreased. What is striking, however, was that the number of cases of depressive disorders was increased in the frailty syndrome group. This may be due to the severity of the disease and its late diagnosis. It is important to note that the frailty syndrome diagnosis has not been standardised. For instance, Sutorius et al. (2016) present discrepancies resulting from the use of different research scales [28]. Many factors influence the development of frailty syndrome. As a result, there are a wide variety of disease presentations. At present, the five criteria that describe frailty syndromes are the most important [29]. The sooner the patient is diagnosed with frailty syndrome, in this case at an early stage, the greater the probability of successful prophylaxis and treatment. Nevertheless, the obtained results indicate that, at the frailty syndrome diagnosis stage, physical training alone, even best adapted to the individual, is insufficient. Consistent with the comprehensive nature of rehabilitation, patients with frailty syndrome should be offered additional forms of support, especially emotional support, so that their condition does not deteriorate. This is even more important considering that our other results show an improvement in lower limb strength in these individuals. These additional forms (e.g., group psychotherapy) are very effective in elderly patients with various chronic diseases [30].

The improvement in the emotional state of the group of patients with pre-frailty syndrome is encouraging and suggests that preventive measures are most effective at this stage of the illness. Therefore, it is worth encouraging patients to start physical activity as early as possible and to perform standard screening tests for depressive disorders. The sooner a patient is diagnosed with the syndrome, in this case at the earlier stage of pre-frailty syndrome, the more effective the prophylaxis and treatment measures.

The difference in emotional status results may also be related to age. The group of patients with pre-frailty syndrome was significantly younger than the group with frailty syndrome. However, many studies have indicated that even the oldest patients still benefit from rehabilitation [25,31].

Changes in the level of anxiety of patients in the studied groups were analogous to mood changes. This is not surprising as anxiety is one of the basic symptoms of depression. This relationship is multidirectional because increased levels of anxiety may intensify the symptoms of depression. The relationship between these characteristics was confirmed by Jaeschke et al. (2010) who described the coexistence of anxiety disorders and depression [32].

Every training programme is considered to be a good way to increase physical fitness among the elderly. People with frailty syndrome experience the so-called “cycle of frailty”, associated with reduced energy expenditure due to insufficient activity. One of the elements that is known to improve the functioning of the elderly and reduce the risk of falls is maintaining an appropriate level of muscle strength, especially in the lower limbs. Studies indicate that resistance training increases muscle strength, walking speed and reduces pain [10,33]. Resistance training using a Thera-band and fitness balls was used in the present research. In our study, systematic physical activity significantly improved the strength of the lower limbs.

High intensity resistance training can be effective in the fight against muscle weakness and decreased fitness in elderly patients. Furthermore, it has been shown that nutritional supplementation without physical activity does not have a significant effect on muscle strength. Beaudart et al. (2017) investigated whether aerobic activity improves VO2 max and muscle strength in the elderly [34]. The procedure consisted of resistance exercises and nine months of gait training, which was found to improve the subjects’ exercise capacity by 14%. It has been demonstrated that multicomponent training, which is one that includes different types of exercises combined with psychological support and patient education, can result in elimination of frailty syndrome by 14.7% relative to a control group [35]. Our training programme included various forms of workouts, including resistance training with the use of equipment, together with aerobic training and stretching exercises. This resulted in improved strength parameters of the lower limbs. In a randomised study where resistance, stretching, neuromuscular control and aerobic exercise were carried out for 24 weeks at 65 min per day, the frailty syndrome elimination was found in 31.4% of elderly people, with no changes in the control group [36]. In our study, training was performed only twice a week, which may not have been sufficient for all results to be statistically significant.

The processes associated with aging include reduced neuromuscular control, reduced muscle strength and cardiovascular diseases; therefore, it is worth trying to prevent these outcomes using endurance and strength training. The research carried out by Cadore et al. (2014) examined the influence of the aforementioned training types on physical fitness of people with frailty syndrome and a control group [37]. Strength training resulted in improvements in muscle strength, speed and ability to recruit motor units. In the second test, endurance training was conducted simultaneously with strength training. It turned out that this combination had a very positive effect on the functionality of the elderly, but it also resulted in a smaller increase in muscle strength compared to strength training alone. Comparing the group of older people to the control group, it was concluded that, when used to improve strength and muscle mass, strength training impacts both groups to the same extent [37]. In our study, the peak torque, total work and average power increased in both groups following the exercise programme.

Other studies revealed that, in elderly women, even short-term strength training (about six weeks) increased the strength of the extensor muscles of knee joint, which also improved their functioning [38]. In our study, exercises were also conducted under the supervision of a specialist, which ensured greater safety and control of the whole training programme.

Research by Tracy et al. (1999) concerned the influence of strength training on the condition of lower limb muscles [39]. An increase in the strength of the dominant limb was observed in both women and men, although it was greater in men. The increase in isometric strength was 13 ± 6% in men and 7 ± 3% in women, but this difference was not statistically significant. Regarding isokinetic strength, significant changes in peak torque of the knee extensors were achieved, but only in the male group. Additionally, the volume of the thigh quadriceps muscle of the trained limb was measured, and a 12% increase was recorded. In our study, we also investigated peak torque in addition to the total work and average power of muscles acting on the knee joint. As a result of the training sessions, these parameters increased in both groups. In terms of percentage, greater differences were observed in subjects with frailty syndrome for most of the parameters. This may be due to the greater muscle weakness observed in this group, evidenced by significantly lower values of strength parameters recorded during the initial study in comparison to the pre-frailty group. The workload for subjects with increased sarcopenia was relatively higher, which resulted in a greater increase in strength capacity of the examined muscles. Higher relative training load of the group of subjects with frailty syndrome may also explain the lack of improvement in mood, or even a slight increase in anxiety and depression.

A study by Batista et al. (2014) examined how the strength of the lower extremities affects independence among elderly outpatients in relation to gender, age and the frailty syndrome criteria [40]. It was noted that men over 80 years of age who met one or two frailty criteria and had greater lower limb strength showed better independence compared to women who were slightly younger but with three or more syndrome criteria. It was also found that men and the elderly with greater strength in the lower extremities had better results with regard to their independence.

Each and every physical activity has a positive impact on the body, evidenced by the improvement in strength parameters observed in both groups investigated in this study. Individuals with diagnosed frailty or pre-frailty syndrome can, with the help of appropriate training, prevent the development of symptoms. This is also important as we observed a simultaneous improvement in the emotional state of patients with a diagnosis of pre-frailty syndrome. On the other hand, deterioration of the emotional state and increased number of cases of depressive disorders were observed among patients with frailty syndrome following training, suggesting that other forms of support and emotional state therapy should be introduced at the same time.

## 4. Limitations

Screening tests were used to assess mood and anxiety in subjects, which is not equivalent to a medical diagnosis and requires more extensive diagnostic tests. The study should be continued with bigger sample size and with the control group of patients. The functional status of patients with frailty syndrome made it necessary for them to be brought to the training sessions by informal caregivers, which could be a stressful factor for the respondents. Further research should consider organising free transportation of patients to the training sessions.

## 5. Conclusions

1. In individuals with pre-frailty and frailty syndrome, the 3-month physical training programme improved the strength parameters of lower limb muscles.
2. An improvement in mood and reduction in depressive symptoms was only observed in the group of subjects with pre-frailty syndrome.
3. Rehabilitation programmes for people with frailty syndrome should include psychotherapeutic activities in addition to physical training in order to improve the psychophysical condition of patients.

## Figures and Tables

**Table 1:** Characteristics of the groups.

	PFS Group *n* = 19			FS Group *n* = 17			Test T		Cohen’s d
	Mean	Median	SD	Mean	Median	SD	*t*	*p*	
Age (Years)	69.16	67.00	5.01	75.35	73.00	6.40	−3.25	0.0026 *	1.12
Height (cm)	153.97	162.00	37.68	149.91	160.00	39.05	0.32	0.7528	0.11
Body mass (kg)	77.05	76.00	11.99	70.35	70.00	14.16	1.54	0.1336	0.53
BMI (kg/m^2^)	29.39	29.00	3.68	27.98	27.34	5.30	0.94	0.3543	0.32

PFS—pre-frailty syndrome group, FS—frailty syndrome group, * Differences were considered statistically significant at *p* < 0.05.

**Table 2:** Percentage distribution of Beck Depression Inventory (BDI) results in the study groups.

Group	BDI Results	T1		T2		Chi^2^ *p*-Value
		*n*	%	*n*	%	
**PFS**	No depression BDI < 12	9	47	15	79	0.0328 *
	Depression BDI ≥ 12	10	53	4	21	
**FS**	No depression BDI < 12	11	65	7	41	0.6275
	Depression BDI ≥ 12	6	35	10	59	

PFS—pre-frailty syndrome group, FS—frailty syndrome group, BDI—Beck Depression Inventory. * Differences were considered statistically significant at *p* < 0.05.

**Table 3:** Comparison of initial (T1) and final (T2) results in both groups (PFS and FS).

Group		T1			T2				T1 vs. T2	
		Median	Min	Max	Median	Min	Max	Wilcoxon Test		r_c_
								*Z*	*p*	
**PFS**	SWLS	22.00	9.00	30.00	23.00	10.00	30.00	0.00	0.5228	0.15
	BDI (1–13)	5.00	0.00	13.00	3.00	0.00	12.00	2.77	0.0058 *	0.77
	BDI (14–21)	4.00	0.00	11.00	3.00	0.00	10.00	2.41	0.0413 *	0.55
	BDI all	12.00	0.00	20.00	5.00	0.00	21.00	2.07	0.0031 *	0.76
	STAI X-1	44.00	26.00	73.00	40.00	26.00	75.00	1.84	0.0079 *	0.61
	STAI X-2	44.00	25.00	74.00	41.00	23.00	53.00	2.07	0.0070 *	0.70
**FS**	SWLS	24.00	20.00	30.00	24.00	13.00	28.00	0.49	0.1075	0.39
	BDI 1–13	2.00	0.00	16.00	5.00	0.00	12.00	0.00	0.2934	0.27
	BDI 14–21	5.00	1.00	12.00	8.00	0.00	13.00	1.46	0.0976	0.40
	BDI all	7.00	1.00	21.00	14.00	1.00	22.00	1.34	0.2093	0.34
	STAI X-1	37.00	26.00	55.00	40.00	23.00	56.00	−0.25	0.4229	0.20
	STAI X-2	38.00	25.00	61.00	40.00	25.00	56.00	1.94	0.1488	0.35

PFS—pre-frailty syndrome group, FS—frailty syndrome group, SWLS-Satisfaction with Life Scale; BDI-Beck Depression Inventory; STAI-Spielberg’s State-Trait Anxiety Inventory; r_c_—pairs rank biserial correlation coefficient; * Differences were considered statistically significant at *p* < 0.05.

**Table 4:** Strength characteristics of the knee joint flexor and extensor muscles at a load of 60°/s, recorded before (T1) and after (T2) the 12-week training programme in both study groups (PFS and FS).

Group		T1			T2				T1 vs. T2	
		Mean	CI	SD	Mean	CI	SD	Student’s *t*-Test		Cohen’s d
								*t*	*p*	
PFS	PT E 60 R (Nm)	97.25	83.56–110.94	27.5	106.19	89.13–123.25	35.4	−0.97	0.3446	0.29
	PT E 60 L (Nm)	92.81	77.80–107.82	31.1	98.28	83.83–112.74	30.0	−2.20	0.0413 *	0.18
	PT F 60 R (Nm)	42.97	35.47–50.47	15.1	50.97	42.40–59.55	17.8	−3.13	0.0062 *	0.50
	PT F 60 L (Nm)	45.52	37.91–53.12	15.8	50.86	42.35–59.37	17.7	−3.19	0.0051 *	0.33
	TW E 60 R (J)	440.89	379.77–502.02	122.9	513.22	436.71–589.72	158.7	−2.39	0.0284 *	0.52
	TW E 60 L (J)	423.96	375.20–472.71	98.0	467.03	394.30–539.76	150.9	−0.92	0.3685	0.35
	TW F 60 R (J)	222.12	184.32–259.91	78.4	258.24	213.58–302.90	92.7	−2.99	0.0078 *	0.43
	TW F 60 L (J)	237.64	195.78–279.50	86.8	260.99	216.14–305.84	93.0	−2.56	0.0196 *	0.27
	aP E 60 R (W)	61.04	51.89–70.20	18.4	64.02	54.00–74.04	20.8	−0.26	0.7993	0.16
	aP E 60 L (W)	58.44	49.24–67.63	19.1	61.52	51.10–71.93	21.6	−1.11	0.2800	0.16
	aP F 60 R (W)	27.63	22.14–33.13	11.0	31.04	25.17–36.91	12.2	−1.58	0.1337	0.30
	aP F 60 L (W)	29.40	23.58–35.22	12.1	31.25	25.28–37.23	12.4	−1.13	0.2728	0.16
FS	PT E 60 R (Nm)	69.35	53.56–85.13	30.7	77.68	61.53–93.82	31.4	−2.91	0.0103 *	0.28
	PT E 60 L (Nm)	68.65	52.86–84.44	30.7	75.31	57.09–93.53	35.4	−2.97	0.0090 *	0.21
	PT F 60 R (Nm)	33.36	25.52–41.20	15.2	39.35	30.28–48.41	17.6	−3.01	0.0083 *	0.37
	PT F 60 L (Nm)	30.86	22.70–39.02	15.9	36.75	28.11–45.39	16.8	−4.98	0.0001 *	0.37
	TW E 60 R (J)	369.06	269.86–468.25	192.9	395.15	308.36–481.94	168.8	−0.98	0.3410	0.15
	TW E 60 L (J)	346.18	259.83–432.53	167.9	373.01	280.36–465.66	180.2	−1.83	0.0861	0.16
	TW F 60 R (J)	168.82	118.11–219.52	98.6	185.09	126.74–243.44	113.5	−0.88	0.3923	0.16
	TW F 60 L (J)	154.66	103.93–205.40	98.7	176.48	129.61–223.35	91.2	−2.40	0.0287 *	0.24
	aP E 60 R (W)	42.33	32.18–52.48	19.7	46.41	36.35–56.47	19.6	−2.25	0.0387 *	0.21
	aP E 60 L (W)	41.10	31.10–51.10	19.4	45.49	34.24–56.75	21.9	−3.04	0.0077 *	0.22
	aP F 60 R (W)	18.83	13.55–24.11	10.3	25.48	19.51–31.44	11.6	−2.56	0.0211 *	0.63
	aP F 60 L (W)	16.83	11.41–22.24	10.5	20.80	15.36–26.24	10.6	−4.47	0.0004 *	0.39

PFS—pre-frailty syndrome group; FS—frailty syndrome group; PT-peak torque; TW-total work; aP-average power; E-knee extensors; F-knee flexors; R-right side; L-left side. * Differences were considered statistically significant at *p* < 0.05; CI—confidence interval ±95%.

**Table 5:** Statistically significant differences in the parameters between the FS group and PFS group in the initial (T1) and final (T2) tests.

Parameters	PFS vs. FS	T1		T2		PFS vs. FS	T1	T2
		U/*t*	*p*	U/*t*	*p*			
SWLS	Mann–Whitney U test	111.00	0.1131	160.00	0.9747	r_rb_	−0.31	−0.01
BDI (1–13)		126.50	0.2743	104.50	0.0734		0.22	−0.35
BDI (14–21)		148.00	0.6804	91.00	0.0265 *		−0.08	−0.44
BDI all		156.50	0.8866	95.00	0.0365 *		0.03	−0.41
STAI X-1		104.00	0.0709	138.50	0.4759		0.36	−0.14
STAI X-2		103.50	0.0685	143.00	0.5684		0.36	−0.11
PT E 60 R	Student’s *t*-test	2.83	0.0078*	2.54	0.0157 *	Cohen’s d	0.78	0.87
PT E 60 L		2.34	0.0254 *	2.11	0.0426 *		1.10	0.72
PT F 60 R		1.88	0.0696	1.97	0.0576		0.81	0.68
PT F 60 L		2.77	0.0089 *	2.45	0.0196 *		1.75	0.84
TW E 60 R		1.32	0.1954	2.16	0.0377 *		0.37	0.74
TW E 60 L		1.69	0.1014	1.70	0.0976		0.99	0.59
TW F 60 R		1.80	0.0801	2.13	0.0407 *		0.76	0.73
TW F 60 L		2.68	0.0112 *	2.75	0.0096 *		1.38	0.94
aP E 60 R		2.90	0.0065 *	2.61	0.0134 *		0.85	0.90
aP E 60 L		2.70	0.0108 *	2.21	0.0341*		0.88	0.76
aP F 60 R		2.44	0.0203 *	1.40	0.1708		0.97	0.48
aP F 60 L		3.31	0.0022 *	2.70	0.0106		2.83	0.93

PFS—pre-frailty syndrome group; FS—frailty syndrome group; SWLS-Satisfaction with Life Scale; BDI-Beck Depression Inventory; STAI-Spielberg’s State-Trait Anxiety Inventory; PT-peak torque; TW-total work; aP-average power; E-knee extensors; F-knee flexors; R-right side; L-left side; rrb—Glass rank-biserial correlation coefficient; * Differences were considered statistically significant at *p* < 0.05.

## Abbreviations

PFS	pre-frailty syndrome group
FS	frailty syndrome group
SWLS	satisfaction with life scale
BDI	Beck depression inventory
STAI	Spielberg’s state-trait anxiety inventory
PT	peak torque
TW	total work
aP	average power
E	knee extensors
F	knee flexors
R	right side
L	left side
T1	initial results
T2	final results
